# Understanding the Folding-Function Tradeoff in Proteins

**DOI:** 10.1371/journal.pone.0061222

**Published:** 2013-04-12

**Authors:** Shachi Gosavi

**Affiliations:** National Centre for Biological Sciences, Tata Institute of Fundamental Research, Bangalore, India; Weizmann Institute of Science, Israel

## Abstract

When an amino-acid sequence cannot be optimized for both folding and function, folding can get compromised in favor of function. To understand this tradeoff better, we devise a novel method for extracting the “function-less” folding-motif of a protein fold from a set of structurally similar but functionally diverse proteins. We then obtain the β-trefoil folding-motif, and study its folding using structure-based models and molecular dynamics simulations. CompariA protein sequence serves two purpson with the folding of wild-type β-trefoil proteins shows that function affects folding in two ways: In the slower folding interleukin-1β, binding sites make the fold more complex, increase contact order and slow folding. In the faster folding hisactophilin, residues which could have been part of the folding-motif are used for function. This reduces the density of native contacts in functional regions and increases folding rate. The folding-motif helps identify subtle structural deviations which perturb folding. These may then be used for functional annotation. Further, the folding-motif could potentially be used as a first step in the sequence design of function-less scaffold proteins. Desired function can then be engineered into these scaffolds.

## Introduction

A protein sequence serves two purposes: it facilitates folding to a stable three-dimensional shape and it provides appropriate residues for binding and activity [1]–[9]. This sequence and its interactions with the solvent define the energy landscape [10] which governs all protein dynamics, both small functional vibrations and large motions like folding [11]–[12]. Thus, it is likely that folding and functional dynamics are coupled and that functional residues affect folding. There has been mounting evidence that functional residues (residues that are part of active sites, binding sites, signal sequences etc.) are a hindrance to stability [13]–[14] and folding [9]. Early folding studies on the WW domain showed that folding rates can be increased at the expense of function [15]–[17]. Since then, the folding-function tradeoff has been observed in several proteins [18]–[26].

The reason for this trade-off is as follows: In order to function correctly, a structured protein has to have specific residues displayed in specific positions over its fold. This imposes two constraints on the protein, that the fold be stable and attainable on a biologically reasonable timescale and that the functional residues be conserved (and not optimized for folding). Only those residues whose chemical and physical properties do not contribute to function can be chosen to make the energy landscape better for folding. Thus, segments containing functional residues are likely to be the hardest to fold [9], [15], [18], [27]. Appropriate mutations to such residues can make folding more efficient but at the cost of protein function [17], [19]–[20].

Functional residues can affect folding either by creating unstable energetic interactions with nearby residues (energetic trapping) or by increasing the complexity of the fold (topological trapping). Energetic trapping has been detected by calculating the ability of mutations to create better local packing than that in the wild-type (WT) protein [27]–[28]. We have previously shown that the topological trapping in the β-trefoil protein [29], interleukin-1β (IL-1β) [30], causes unfolding and refolding of partially formed structures along its folding route [31]. This ‘backtracking’ is caused by the interactions between two distal loops which make up a binding site of IL-1β. Both computationally [18] and experimentally [19], mutating the binding site loops reduces backtracking and increases the folding rate.

The case of IL-1β indicates that the fastest folding protein might be achieved by removing all functional sites in the protein. In order to test this hypothesis and better understand the effects of function on the folding of WT proteins, we create a computational model of the “function-less” folding motif (FM) of the β-trefoil fold [29]. In proteins which adopt the same structure but have diverse functions and little sequence similarity (e.g. a fold from SCOP [32]), the structurally conserved regions are likely to facilitate efficient folding and stability, while the differences (e.g. binding loops) are likely to be involved in individual function. Here, we develop a method to extract the structurally conserved regions, i.e., the FM, of a structural family of proteins and apply it to the β-trefoil fold. The construction of the FM partitions WT residues into structural (those that structurally align with the FM) and functional (those that have no equivalent residues in the FM) regions similar in spirit to the partitions obtained from protein co-evolution methods [33]. Here, we take this a step further and study the folding of the structural network of residues (FM) to understand how function affects folding.

**Figure 1:**
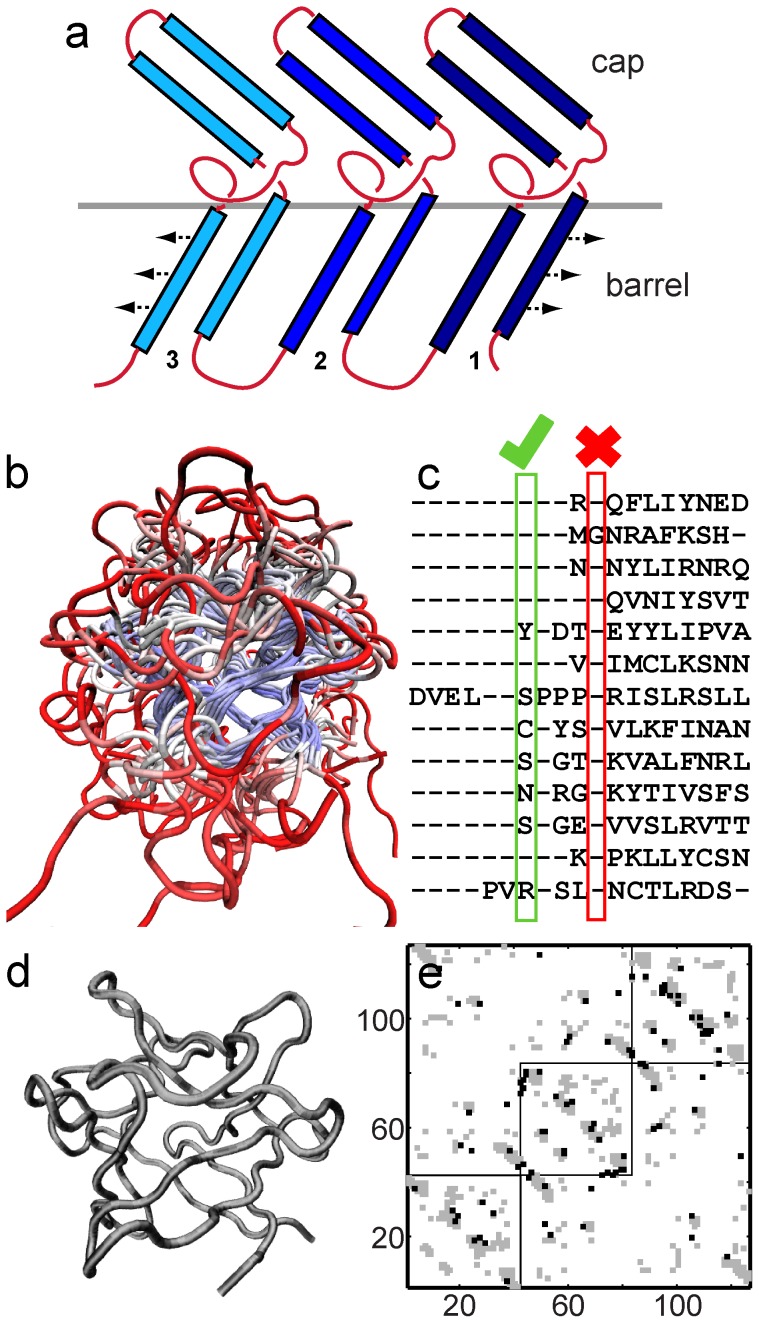
Design of the folding motif (FM). **(a)** Cartoon of the β-trefoil fold. The three pseudo-symmetric trefoil units are shown in different shades of blue. The two edge strands of trefoil 1 and 3 hydrogen bond (represented by arrows) to form the barrel. The red loo"sps vary in length and secondary structural content across different β-trefoil proteins. **(b)** A representative structural alignment of the 13 proteins used for the FM design. The colouring shows the most similar regions (blue) and the least similar regions (red). All protein figures are plotted using VMD [34]. **(b)** Part of the structure alignment derived sequence alignment of the 13 proteins. Each of the lines of sequence shown is from a different protein. A residue position is chosen to be part of the FM if it contains a residue and not a gap in at least 7 of the proteins. A chosen position and a rejected position are shown for illustration. **(c)** The backbone of the FM derived from this construction has 127 residues. The view of the FM is the same as that of the aligned proteins in (a). **(d)** Two slightly different contact maps for the FM. The x and the y axes show the residue index of the FM. If an interaction is present between residues ‘i’ and ‘j’ of the FM then filled boxes are marked on the contact map at (i,j) and (j,i). The first map with fewer contacts (348 in number) is depicted in grey. The second map includes both the grey and the black contacts (totally 395). The three squares enclose the intra-trefoil contacts of the first (N-terminal), second (central) and the third (C-terminal) trefoils and demonstrate the three-fold pseudo-symmetry of the β-trefoil fold. As detailed in the text, the FM maps are derived from the contact maps of the WT proteins and not directly from the FM backbone.

We chose the β-trefoil proteins (Fig. 1a) for this study because their individual binding sites are chemically different, bind diverse molecules including DNA, proteins and carbohydrates and are located in different parts of the fold [29]. Thus, the functional regions will not be structurally conserved across functionally diverse proteins (Fig. 1b) and will not be present in the FM. The β-trefoil fold is composed of 12 β-strands, 6 of which form a hairpin triplet capping a 6-stranded barrel (Fig. 1a). The loops connecting the β-strands are variable in both sequence and length (red regions in Fig. 1a and 1b) and are the cause of the functional variability of the fold [29]. The cap hairpins and their flanking barrel β-strands make up three pseudo-symmetric (β-β-β-loop-β) trefoil units (Fig. 1a and 1e). Here, we ignore the inherent pseudo-symmetry of the fold in order to develop a general method which can be applied to any protein structural family.

This article introduces the idea of one FM for an entire fold. This FM is made “functionless” by optimizing protein length and packing. The folding of the FM is computationally studied using coarse-grained structure based models (SBMs), which have been successfully used to understand and predict transition states, folding routes and folding rates of WT proteins [35]–[37]. The folding of the FM helps us understand the collective folding landscape of the β-trefoil proteins. We then systematically mutate both the FM and WT β-trefoil proteins to understand the compromises in folding and stability that a protein can make in order to conserve function. Specifically, we compare the folding of the FM to that of two WT proteins IL-1β [30] and hisactophilin (HIS) [38]. We choose these proteins for two reasons: (a) Folding experiments [19]–[20], [39]–[43] corroborate the folding routes and rates found in MD simulations [18], [31], [44], [45] of these proteins. These simulations were performed using SBMs similar to those used in this article. (b) Simulations show that IL-1β folds slower than the FM (Fig. 5c) while HIS folds faster (Fig. 6c). We want to understand the structural and functional differences between the WT proteins and the FM which cause these opposing effects on folding rates.

## Methods

### Structure-based models (SBMs)

The timescale of protein function puts an upper bound on the folding time of proteins. The folded state of the protein has to be kinetically accessible from the unfolded ensemble within this time. This is made possible by a funnel-shaped energy landscape where the folded staten perform MD simulations of these S of the protein is at the bottom of the funnel and there is a structural bias towards the native state [46]. This bias implies that local interactions stabilize native-like structure and lead to further folding. The stabilization due to non-native traps is small (∼2 k_B_T) and can be overcome using thermal energy [46]. SBMs (or Gō models) of proteins [35]–[37] take this a step further and ignore all non-native interactions. The energy function within these models encodes only the native or the folded structure of the protein as determined in an X-ray or an NMR structure. This energy function is then used to perform molecular dynamics (MD) simulations [35].

#### The energy function used in the C-α SBM

The specific version of the SBM used in this article contains a coarse-grained description of residues and has a single bead present at the position of the C-α atom of each residue [35]. The functional form for the SBM potential energy function is [31], [35]: 
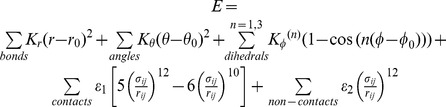
The first three terms represent the energies of bond vibration, angle fluctuation and dihedral rotation respectively and the terms are summed over all the bonds, angles and dihedrals of the backbone. These three energies have their deepest minima at the values *r_0_*, *θ_0_* and *φ_0_* calculated from the from the x-y-z coordinates of the C-α atoms in folded state. *K_r_ = 100ε*, *K_θ_ = 20ε*, *K_φ_^(1)^ = ε* and *K_φ_^(3)^ = 0.5ε* are the force constants of the bond, angle, and the two dihedral angle terms. Thus, the bonds and the angles are much stiffer than the dihedral angles. The final two terms give the through space interaction energies between the C-α atoms. The first of these terms is an attractive Lennard-Jones 10–12 potential between those C-α atoms whose amino acids are in “contact” in the native state. A list of such pairs of C-α atoms, *(i, j)*, defines the “contact” map. *σ_ij_* is equal to the distance between the C-α atoms, *i* and *j*, in the folded state of the protein. The second term defines a short range repulsive interaction between pairs of C-α atoms not in contact. This term ensures that C-α atoms not in contact do not pass through each other during the dynamics. *σ* is defined to be *4.0 Å*. *ε_1_ = ε_2_ = ε*. The basic energy scale in our simulations, *ε*, is equal to 1 kcal/mol.

### MD simulations of SBMs

The inputs to the SBM are (a) the coordinates of each C-α atom and (b) a list of the interactions between these atoms, the contact list (which defines the contact map). For WT proteins, the coordinates are extracted from the corresponding pdb files and the contact list is generated from the pdb file using CSU analysis [47]. We use the sander_classic program of the AMBER5 package [48] to perform all MD simulations. The free energies of the folded and the unfolded ensembles are equal at the folding (or melting) temperature, T_f_. If the folding barrier is small, then the protein transitions between the two ensembles multiple times and this ensures adequate sampling of the transition region. β-trefoil proteins are slow-folding and not accessible to normal constant temperature MD simulations [31]. To acquire adequate sampling near T_f_, we use a previously developed modified multicanonical method [31]. This method enhances sampling in the transition region by rescaling the normal MD force by a Gaussian weight. The resulting sample is then reweighted to recover the usual (NVT) distribution. We next outline the method used to generate the coordinates of the C-α atoms and the contact map of the FM from the structures and the contact maps of 13 WT β-trefoil proteins.

### Choosing the residues of the FM using a structural alignment of a set of functionally diverse proteins from the β-trefoil fold

The SCOP database [32] classifies proteins into different folds. Within these folds, proteins from different families “have related sequences but distinct functions” [32]. One protein is picked at random from each of the 13 families of the β-trefoil fold included in the database. The Multiseq extension [49] (the STAMP algorithm [50]) of VMD [34] is then used to create a structural alignment of the chosen proteins. The pdb IDs of the proteins, the total number of residues and the number of calculated contacts (if a specific chain or a specific set of residues from the pdb file are used, then this information is appended at the end) are: 2AFG (129∶374:chain-A), 6I1B (153∶430), 1T9F (178∶532), 1SR4 (154∶367:chain-C), 1DQG (134∶388), 1UPS (131∶380: chain-A:290–420), 1JLY (153∶457:chain-A:1–153), 1WBA (171∶499), 1DFQ (193∶528: 1123–1315), 1DFC (119∶347:chain-A:1141–1259), 1HCD (118∶324), 1TTU (161∶428: chain-A:381–541), 1WD4 (162∶481:338–499).

**Figure 2:**
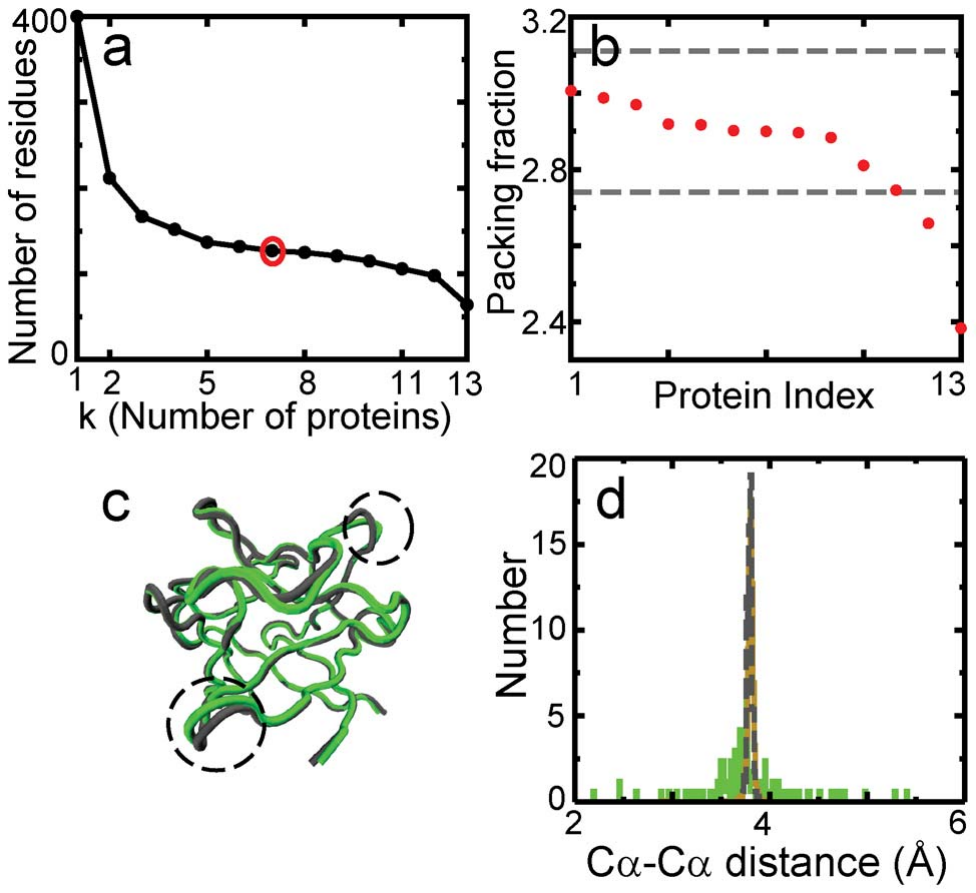
Picking residues and contact maps for the FM. **(a)** The number of residue positions (see Fig. 1c) that would be in the FM is plotted against the minimum number of proteins that must have an amino-acid (and not a gap) in the alignment at each position. In the FM, a residue position is chosen if an amino-acid is present in 7 or more proteins. This is marked with a circle on the plot. Note that the FM occurs in the flat part of the plot. This means that the number of chosen residues changes by little if the minimum number of proteins is changed by ±1, and the choice of 7 does not make a large difference to the FM. **(b)** The packing fraction (total number of contacts/the total number of residues) for the 13 proteins used to create the FM is sorted in descending order and marked by red circles. The two contact maps chosen for the FM (Fig. 1e) have packing fractions slightly above and below that of the median of the 13 proteins and these are marked by grey dashed lines. **Optimizing Cα-Cα distance in the FM.** **(c)** A structural alignment of the FM before (green) and after (grey) optimization. The dashed circles show differences in loops that are clearly visible. Overall, the largest changes occur in the loop regions. **(d)** Normalized histograms of Cα-Cα distances: The histogram of the Cα-Cα distances from the 13 proteins used to create the FM is shown in brown. In green is the histogram of Cα-Cα distances from the FM before optimization (green structure in (a)). In dashed grey is the histogram of the FM distances after optimization (grey structure in (a)).

The structural parts of these proteins overlay well (Fig. 1b and 1c) and residues from regions which are common to a majority of the proteins are selected to be part of the FM (Fig. 1c and 1d). A given protein either has an amino-acid or a gap at each position of the alignment. A position or a “residue” is chosen to be part of the FM if more than 50% of the proteins in the alignment (here 7 or more) have an amino-acid and not a gap at that position (Fig. 1c). Figure 2a shows a plot of the number of residues (on the y axis) that are aligned in ‘k’ or more proteins (on the x axis). This plot is flat around 7 and the number of residues which get chosen for the FM is not sensitive to the exact value of 50%.

**Figure 3:**
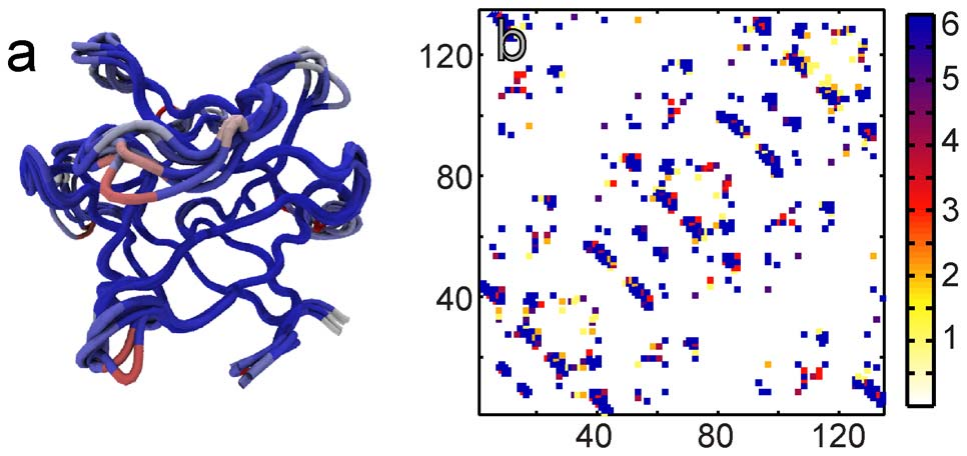
Variability among FMs. **(a)** An alignment of the FM backbone (Fig. 1d) with the backbones of 5 other β-trefoil FMs generated using 5 different sets of 13 WT proteins. The most similar regions are shown in blue while the least similar ones are shown in red. Most of the barrel and cap β-strand C-α atoms are so structurally conserved that the aligned backbones merge and only a single backbone can be observed (for comparison, see Fig. 1b and 1d). The differences between the FMs lie mainly in the loop and turn regions. **(b)** A composite of the contact maps of the 6 backbones. On the x and the y axes, the residues are numbered according to their order in the structural alignment shown in 3a. This order is calculated from an output similar to the one shown in Fig. 1c. The colour of a contact indicates the number of FMs that the contact is common to.

One can imagine an anti-parallel β-barrel fold whose proteins structurally align well. Despite this if the length of each β-strand is very variable across the proteins then the number of residues common to ‘k’ proteins could decrease continuously as k increases. The length of the FM would then be sensitive both to the number of proteins included in its construction and the threshold for picking residues (50% for the present FM). Whether such folds exist in nature needs to be tested, but the β-trefoil is not such a fold. The number of residues common to k or more proteins drops to below 150 by k = 4 and stays at above 100 even when k = 12 (Fig. 2a). The final drop in number of residues between k = 12 and k = 13 occurs because one of the 13 proteins has only 2 cap hairpins (Fig. 1a). Furthermore, five different FMs have very similar β-strand C-α coordinates and vary in length from the simulated FM by only ±3 (Fig. 3a). Thus, a canonical structure of the β-trefoil fold (in terms of geometric factors such as length of individual β-strands) can be extracted and is captured by the FM.

We also test that adjacent C-α atoms in the FM are connected to each other in at least one of the WT proteins. In the present FM, this is indeed true. If adjacent FM C-α atoms are not connected in any of the WT proteins, then it is possible that the corresponding aligned regions are very variable across the WT proteins. The FM method will not select enough residues from such regions. A possible solution to this problem is to pick the shortest of the WT backbones aligned to the FM in the variable region and locally build an FM backbone based on it.

The initial x-y-z coordinates for a “residue” (or position) chosen to be part of the FM are calculated by averaging over the x-y-z coordinates of the C-α atoms of the residues from all the proteins which have a residue (and not a gap) at that position. This averaging is possible because Multiseq [49] overlays the proteins and outputs pdb files with the aligned coordinates. Since these coordinates are averages they do not satisfy C-α-C-α distance constraints of the β-trefoil proteins. We use MD to make appropriate modifications to the FM backbone, as described in a later section. The final FM backbone is shown in Fig. 1d.

### Choosing the contact map for the FM

The contact map for the FM is derived from the contact maps of the WT proteins. A contact between FM residues ‘i’ and ‘j’ is chosen to be part of the FM contact map, if the contact is present between the corresponding aligned residues (no matter what the identity of the residues is) in a sufficient number of the aligned WT proteins (here either ≥4 or ≥3). We choose this threshold such that the FM has a ratio of number of contacts to number of residues similar to that of WT proteins. We call this ratio the packing fraction. If the packing fraction is too small in the FM then there are not enough contacts per residue and the protein is too loosely packed. If the packing fraction is too high then a residue might be making a physically impossible number of contacts. Any given atom can be surrounded by or be “in contact” with only a limited number of atoms. For a given atom, this limit will depend both on its own size and on the size of the atoms surrounding it. In the C-α model, the contacts of all the atoms of a given residue are assigned to its C-α atom. Thus, there is a physical limit on how many “contacts” a C-α atom can make. This number will depend not only on the sizes of the component atoms and how well they are packed but also on how big the residue is. Thus fixing the packing fraction is akin to requiring a level of residue packing which is appropriate for the fold.

We plot the packing fraction for the chosen WT proteins and the two FM contact maps in Fig. 2b. The map with the higher packing fraction (∼3.1) has more contacts and was calculated using contacts which were common to 3 or more WT proteins. The other map (packing fraction ∼2.7) was calculated using contacts which were common to 4 or more WT proteins. We choose these contact maps because their packing fractions are closest to the median packing fraction (∼2.9) of the WT proteins. The chosen contact maps corresponding to the two thresholds are given in Fig. 1e.

We note in passing that the backbone and the contact maps of the FM show a higher degree of three-fold symmetry than those of the WT proteins.

### Fixing the FM bond-distance distribution

The FM created using average x-y-z coordinates does not have C-α-C-α bond distances appropriate for the β-trefoil fold. The distance distribution for the WT β-trefoil proteins is given in Fig. 2d and is sharply peaked around 3.8 Å. We modify the FM to make its C-α-C-α bond distance distribution closer to that of the WT proteins. We start with the standard SBM potential given earlier but modify all the bond length parameters (*r_0_*) in this potential to 3.8 Å. We then increase the dihedral (*K_φ_^(1)^* and *K_φ_^(3)^ = 0.5 K_φ_^(1)^*) and contact (*ε_1_*, *ε_2_*) force constants to be the same as the angle force constants (*K_θ_ = 20ε*). We perform a short MD simulation using this modified potential. Upon simulation we find that the C-α atoms in the loop regions (Fig. 2c) relax to give an appropriate bond distance distribution (Fig. 2d). The positions of the C-α atoms in the core of the protein remain largely unaltered.

### Reaction coordinate

Since SBMs are based on contact maps, the fraction of contacts that are formed (Q) gives us a measure of how folded a protein is and we use Q as a reaction coordinate. Q is commonly used in SBM simulations [31], [35]. To examine the progress and the mechanism of folding we plot various quantities: the probability of contact formation for all contacts (a probability coloured contact map), the scaled free energy (ΔG/k_B_T_f_), etc. as functions of Q.

**Figure 4:**
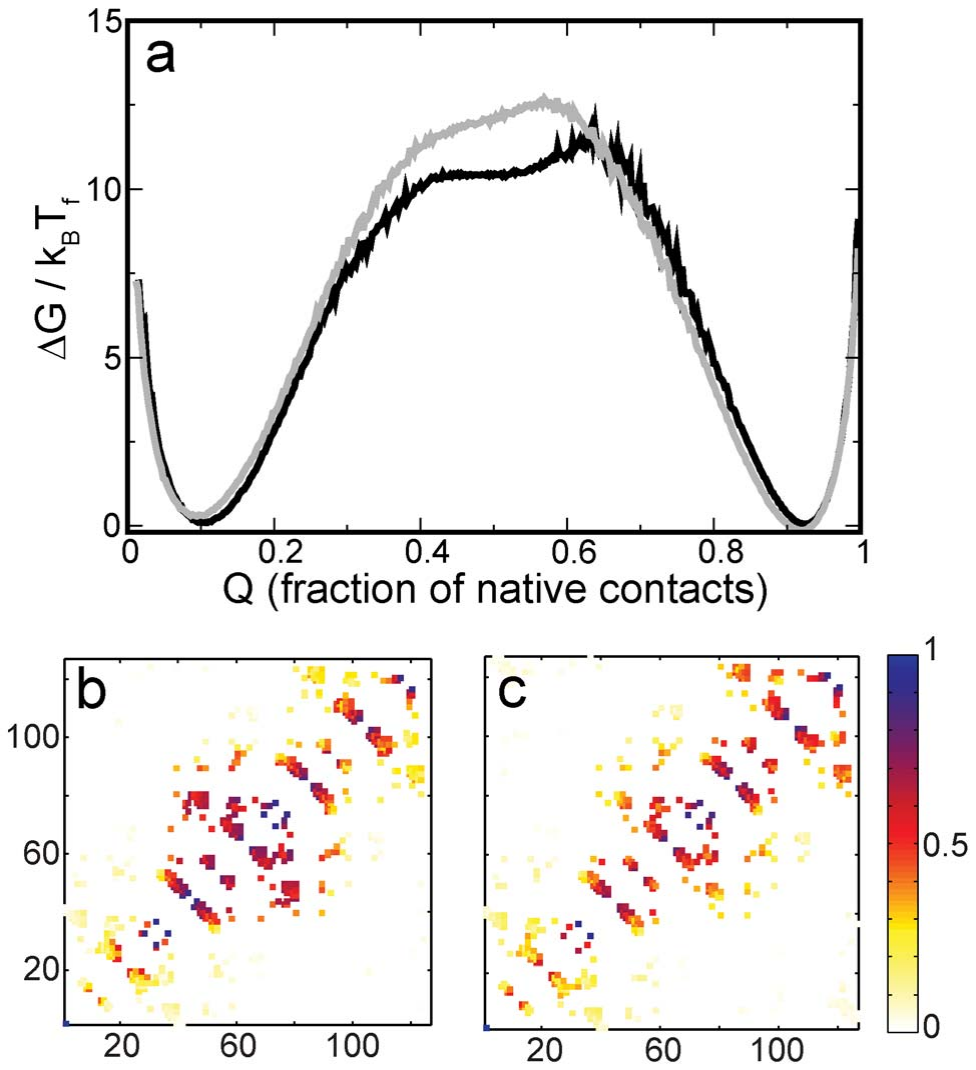
Folding barriers and routes for the FM. **(a)** Free energy profiles (in scaled units) of the FM for the two contact maps shown in Fig. 1e plotted as a function of the fraction of native contacts. The profile for the grey contact map is in grey. The profile for the grey+black contact map is in black. Although the profiles have different barrier shapes the maximal heights of both are almost the same. **(b)** Average contact map associated with the black free energy profile when that protein is 45% folded or Q = 0.45. The colour bar provides a measure of how formed a contact is on average, with one indicating completely formed and zero not formed. **(c)** Average contact map associated with the grey free energy profile when Q = 0.45. (b) and (c) illustrate the change in dominant folding route upon altering the contact map. The specific value of Q = 0.45 is chosen because it best differentiates between the folding routes.

**Figure 5:**
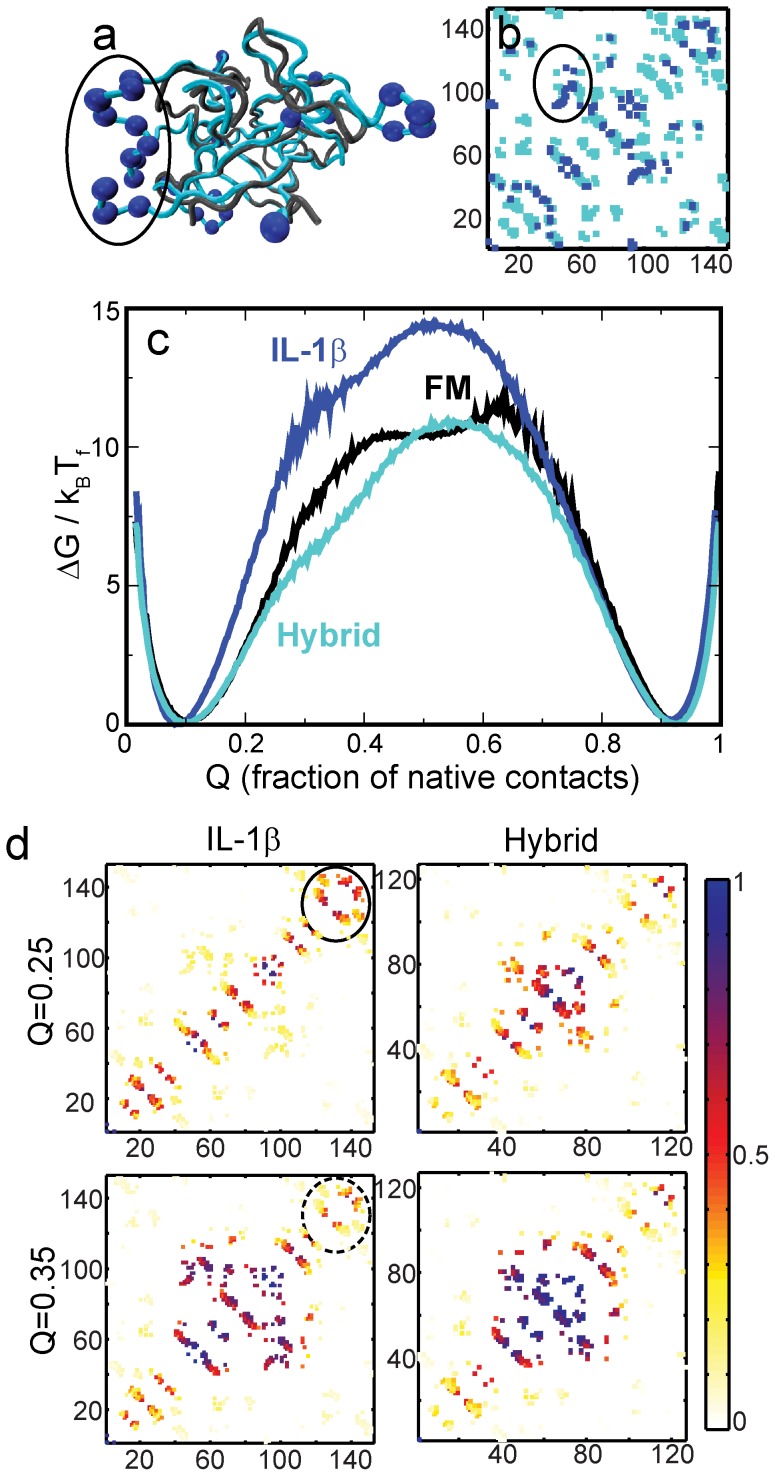
Binding sites make folding more complex in IL-1β. **(a)** Structural alignment of the FM (grey) and IL-1β (6I1B; cyan). Several loops of IL-1β are longer and more structured than those of the FM. Residues present only in IL-1β are marked by blue spheres. These residues correspond well with known binding sites of IL-1β [54]–[56]. The circled residues show the B-binding site. Removing this site reduces backtracking [18]. **(b)** Contact map of IL-1β projected onto the FM (contacts of IL-1β between residues which have a corresponding aligned residue in FM) is marked in cyan. Rest of the IL-1β contacts are marked in blue. The circled blue contacts are part of the B-binding site and are absent in the hybrid-IL-1β. Residue numbering is that of IL-1β. **(c)** Free energy profiles of IL-1β (blue), FM (black) and the hybrid (FM backbone + cyan contact map; cyan). Although the shapes of the barriers are different, the barrier height of the hybrid profile is almost the same as that of the FM. **(d)** Average contact maps of IL-1β (cyan backbone in (a); blue and cyan contacts in (b); blue free energy profile in (c)) and of the hybrid-IL-1β (grey backbone in (a); cyan contacts in (b); cyan free energy profile in (c)) at Q = 0.25 and Q = 0.35, respectively. The circled contacts in IL-1β form early but are not present when the protein is 35% folded. These contacts show the primary region of backtracking. There is little backtracking in hybrid-IL-1β. As in Fig. 4b and 4c, the colour bar provides a measure for how folded a contact is on average. The values of Q = 0.25 and Q = 0.35 are chosen because they best illustrate the change in backtracking between the two proteins.

**Figure 6:**
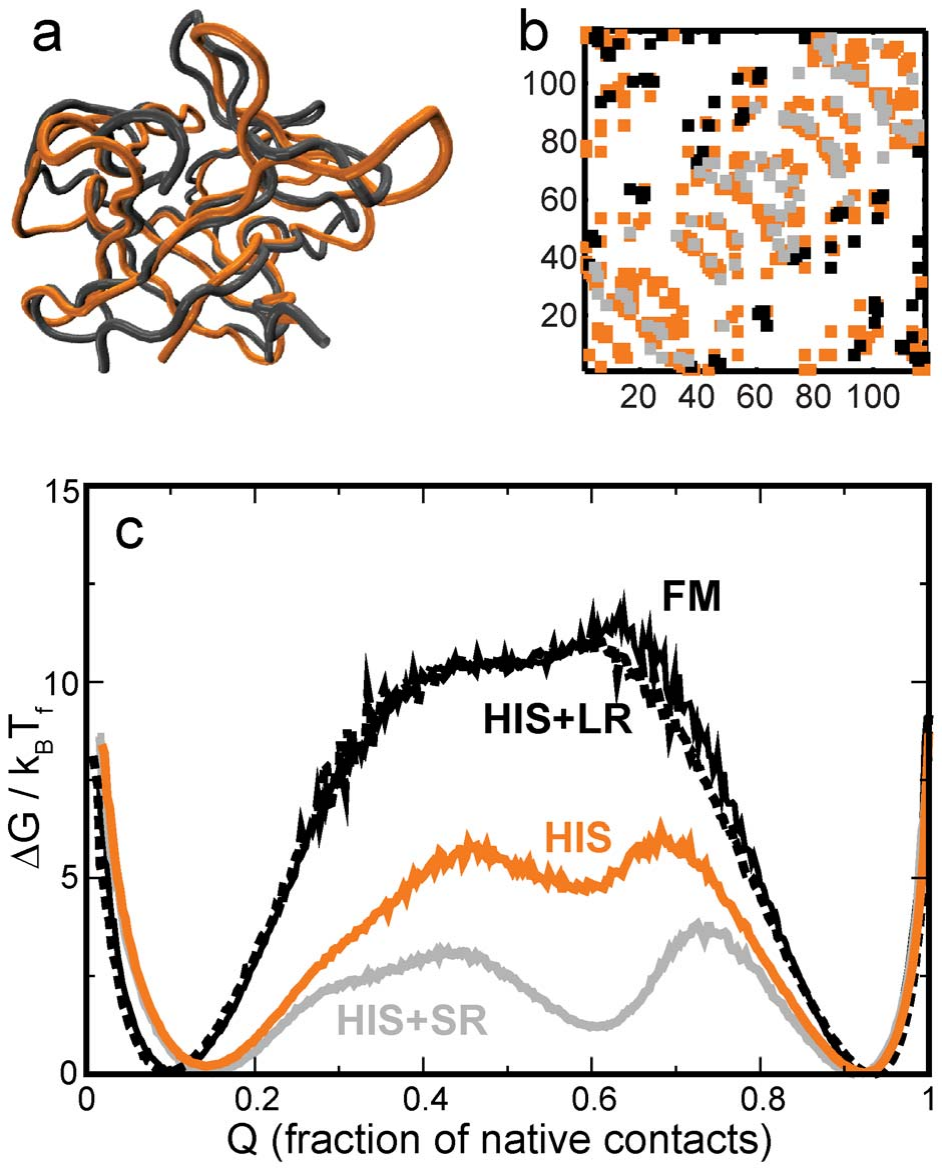
A comparison of the structure and folding of hisactophilin (HIS) with that of the FM. **(a)** Structural alignment of the FM (grey) and HIS (1HCD; orange). HIS is shorter than the FM and, except for the loop seen on the top right, loops of HIS are shorter than those of FM. **(b)** Contact map of HIS shown in orange. FM contacts projected onto the HIS backbone are shown in grey and black. Short-ranged (SR) contacts (with short loop lengths [58]) present only in the FM are shown in grey. Long-ranged contacts present only in the FM are shown in black. Contacts common to both HIS and FM are part of the orange HIS contact map and not shown separately. **(c)** Free energy profiles of the HIS backbone with different contact maps. The HIS+SR protein has the orange and grey contacts from (b) while the HIS+LR (black dashed line) has the orange and the black contacts from (b). The black contacts from (b) increase the barrier to folding to the same level as that of FM (black solid line).

**Figure 7:**
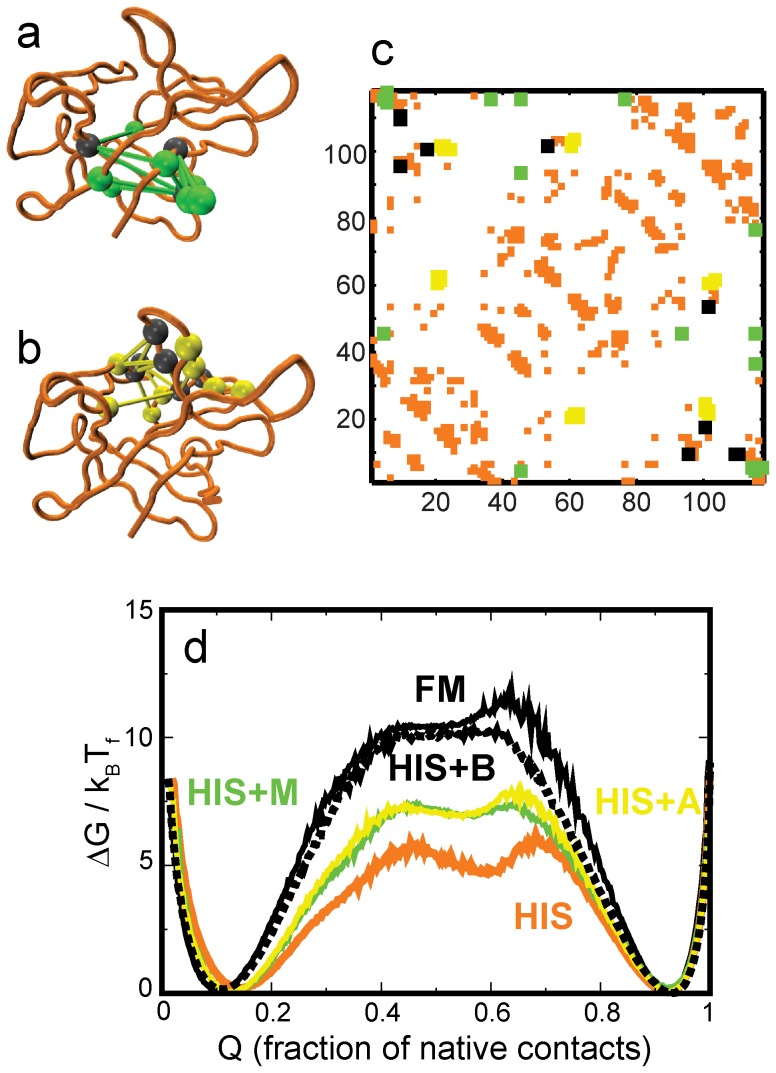
Binding sites decrease the barrier to folding in hisactophilin. **(a)** Structure of HIS (orange) with key myristoyl binding residues [43], [45] marked in grey. In order to accommodate and bind the myristoyl chain the grey residues do not have the contacts marked in green (M) with the green residues. The FM has these contacts. **(b)** Structure of HIS (orange) with a cluster of putative actin binding residues [59] marked in grey. The FM has the contacts between the grey and the yellow residues (marked in yellow; A) while HIS does not have them. **(c)** The HIS contact map (orange) with chosen long-ranged FM contacts (B: green, yellow and black. These contacts are marked at twice the size of the other contacts.). The green contacts denote the myristoyl binding site and are the same as shown in (a). The yellow contacts are the ones shown in (b). The black contacts do not form a structural cluster and we do not use them in independent simulations. Details of how the contacts are chosen are given in the text. **(d)** Free energy profiles of the HIS backbone with different contact maps. The HIS+M protein has the orange and green contacts from (c), the HIS+A protein has the orange and yellow contacts from (c), and the HIS+B protein (black dashed line) has all (orange, green, yellow and black) the contacts shown in (b). The folding barrier of HIS+B is almost as high as that of the FM (black solid line).

Changing contact maps usually changes T_f_. A higher T_f_ means that the protein has more thermal energy (k_B_T_f_) and it is easier to cross the folding barrier. In order to compare folding barriers at different T_f_'s, we scale the barrier heights by the thermal energy. The values of k_B_T_f_ (in units of kcal/mol) for the various proteins simulated in this article are: FM (348 contacts, Fig. 1e and 4a): 1.11, FM (395 contacts, Fig. 1e and 4a): 1.21, IL-1β (Fig. 5c): 1.13, IL-1β-FM hybrid (339 contacts, Fig. 5b and 5c): 1.10; HIS (Fig. 6c): 1.12, HIS+LR (368 contacts, Fig. 6b and 6c): 1.24, HIS+SR (368 contacts, Fig. 6b and 6c): 1.24, HIS+A (337 contacts, Fig. 7c and 7d): 1.16, HIS+M (334 contacts, Fig. 7c and 7d): 1.16, HIS+B (Fig. 7c and 7d): 1.2. The values of k_B_T_f_ for the proteins are close to 1 and even closer to each other. Thus, the scaling changes the results by little.

### Robustness of the FM construct and control simulations

In order to test the robustness of the FM, we constructed 5 additional FMs using 5 different sets of 13 proteins randomly chosen from the different families of the β-trefoil fold. The aligned FMs and their composite contact map are shown in Fig. 3a and 3b, respectively. The length of these FMs changes by only ±3 residues from the length of the FM construct (127 residues) used in the folding simulations. In addition, the coordinates of the aligned β-strand C-α atoms vary by very little between FMs. The variability in the number of residues and the coordinates of aligned residues is largest in the loop regions (Fig. 3a). There are 120 C-α atoms and 290 contacts common to all 6 FMs. This underlines our previous observation that a canonical FM exists for the β-trefoil fold.

A fibroblast growth factor (2AFG.pdb) is part of our set of chosen proteins and is the protein closest in shape to the FM (both in the number of residues and their alignment with the FM). To ensure that the choice of the FM is not biased by the presence of a similar protein in our initial dataset, we constructed another FM using our initial dataset but with no 2AFG. On performing SBM simulations of this construct, we found that the folding routes and barrier heights changed little across the two FMs.

As can be seen from its functional form, the potential energy used in the present SBM depends on the number of contacts and the number of dihedrals present in the folded state of the protein being simulated. The contact to dihedral ratio is the ratio of the number of contacts to the number of dihedrals in the folded state of the protein. At times, a change in the contact to dihedral ratio affects free energy barrier heights [44]. The number of dihedrals equals n-3 where n is the number of residues. So, the contact to dihedral ratio is approximately equal to the packing fraction defined earlier. Although the contact to dihedral ratio of individual proteins varies, it can be seen from the plot of the packing fraction in Fig. 2b that this number lies in a small range for all simulated proteins. Also, where possible, models with the same contact to dihedral ratio have been compared in order to reduce any bias of the variation of this ratio upon the results.

## Results

### Folding properties of the FM

We use the backbone (Fig. 1d) and the two contact maps (Fig. 1e) to derive the two different SBMs of the FM. We then perform MD simulations of these SBMs. Simulations of all proteins are performed close to T_f,_ the temperature at which the folded and the unfolded states are equally likely (see Methods). Thus, all free-energy profiles have folded and unfolded basins of equal free energy. Our simulations are performed using a modified multicanonical method [31] and then reweighted to give the free energy profiles. Because of the use of this technique, simulation time cannot be converted into “real” time, and we use the free energy barrier heights to understand the effect of changing parameters (contact maps and backbones) on the folding rate. A higher barrier height implies a lower folding rate and vice versa [51].

The free energy profiles from the FM simulations are given in Fig. 4a. We find that the difference in contact maps between the two models of the FM does not affect the barrier height significantly (Fig. 4a). However, the models have different dominant folding routes (Fig. 4b and 4c). The central trefoil (see Fig. 1a and 1e) folds first in the model with the larger number of contacts (Fig. 4b). The third (see Fig. 1a and 1e) and the central trefoil fold simultaneously in the model with the smaller number of contacts (Fig. 4c). The contacts between the termini form earlier in this route.

These are the two folding routes seen in experiments on β-trefoil proteins [19]–[20], [39]–[43], [52]–[53]. Thus, the FM encapsulates the entire known folding landscape of the β-trefoil fold [44]. Furthermore, the FM demonstrates that multiple folding routes are intrinsic to the β-trefoil folding landscape and minor perturbations in contact maps can induce a change in the dominant folding route of a β-trefoil protein. Finally, in agreement with current β-trefoil folding experiments [19]–[20], [39]–[43], [52]–[53], the first trefoil does not act as a folding nucleus.

We next compare the folding of the FM to the folding of two WT β-trefoil proteins. We pick the FM with the larger number of contacts for this comparison because it is better packed and better folding (has a lower barrier).

### Comparison with IL-1β

The signaling cytokine, IL-1β, has three known binding sites, A, B and C [54]–[56]. Sites A and B are respectively used to bind and to induce a conformational change in receptor IL-1R1 [54]–[55]. The newly discovered site C binds the IL-1Racp co-receptor [56]. The structure of the receptor blocker IL-1Ra closely resembles that of IL-1β and it binds via site A to IL-1R1. Due to the absence of site B, IL-1Ra cannot induce a conformational change in the receptor and blocks it [57]. Recent simulations [31] and experiments [19] on IL-1β showed the presence of backtracking in the dominant folding route of IL-1β. The backtracking was caused by the presence of site B, and replacing a functional β-bulge in IL-1β by the corresponding smaller non-functional β-turn from IL-1Ra not only reduced the folding barrier but also reduced backtracking significantly [18]–[19]. In the previous work [18], the existence of the closely related IL-1Ra made it possible to understand the role of binding sites in increasing topological trapping and backtracking during folding. Here we reproduce the same results using the FM. The advantage of the FM is that it renders the existence of a closely related protein unnecessary for structural comparison.

We first structurally align [49]–[50] IL-1β and the FM (Fig. 5a) and identify those residues of IL-1β which align with gaps in the FM (marked with blue spheres in Fig. 5a). Most of these residues (1–3, 32–35, 49–50, 52–55, 86–94, 118, 142, 153) lie in the A, B or C binding sites. The FM does not have these loops and thus has fewer binding residues. There are structural additions in IL-1β (residues 40, 118, 75–76, 78) that do not lie around the known binding site regions and we predict that the 75–78 loop might be functionally significant.

There are a few scattered residues in the FM (11, 33–34, 101, 113) which align to gaps in IL-1β but since these are scattered around the fold and do not form a specific structural motif we do not expect them to contribute significantly to either folding traps or function.

We next create a contact map for the FM backbone using only contacts present in IL-1β (Fig. 5b). This is akin to removing the binding loops of IL-1β [18]. The folding of the FM backbone with IL-1β contacts (hybrid IL-1β) is shown in Fig. 5c. The height of its folding barrier is less than that of WT IL-1β. The folding of the hybrid also shows no backtracking (Fig. 5d). Since the hybrid has fewer binding residues and is structurally simpler, we conclude that the binding sites of IL-1β increase the folding barrier and the complexity of folding.

### Comparison with hisactophilin (HIS)

**Figure 8:**
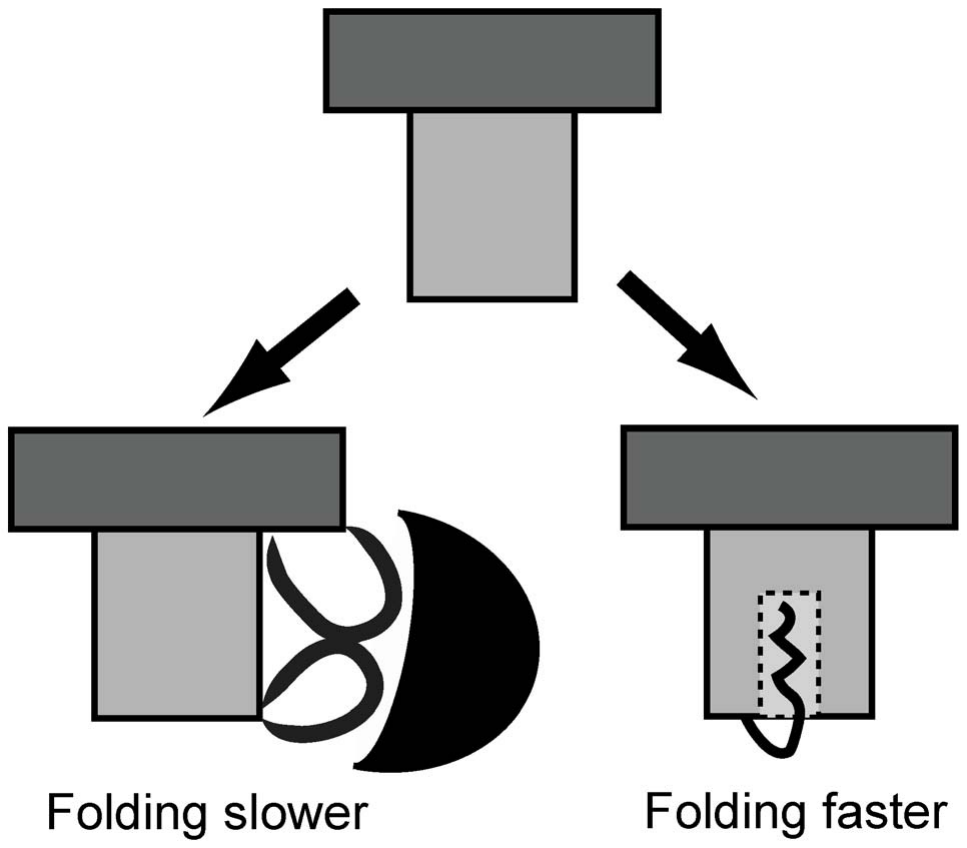
The folding-function tradeoff. Cartoon of an ideal β-trefoil fold (the hairpin triplet cap is shown in dark grey while the barrel is in pale grey) and two ways in which function can be introduced into it. On the left is a representation of what happens in the case of IL-1β, where function is added through extra structural elements. The binding partner is shown as a black crescent. On the right is a cartoon of HIS. Here fold residues are reassigned to create a cavity (dashed square) within the fold. The cavity is used to sequester the N-terminal myristoyl chain.

The slime mold actin and membrane binding β-trefoil protein HIS [38] has a known myristoyl binding site within its β-barrel [43]. HIS is myristoylated at an N-terminal glycine. The myristoyl chain switches between an exposed state which enables membrane binding and a state where it is buried in the β-barrel of the fold (cartoon shown in Fig. 8) [43], [45]. HIS has a lower folding barrier than the FM (Fig. 6c). An alignment of the backbones of the FM and HIS is shown in Fig. 6a.

We construct several hybrid contact maps for the HIS backbone in order to understand the reasons for the lower folding barrier in HIS. Figure 6b shows the HIS contact map (324 contacts) and the 88 contacts which are present in the FM but not in HIS. We order these 88 contacts by their loop-length. (The loop-length of a contact is the number of residues along the protein backbone that separate the two residues that form the contact [58].) We then split the contacts into two groups of 44 contacts: one with lower loop-lengths (SR: short range) and the other with the higher loop-lengths (LR: long range). We construct two hybrid HIS models, one with the HIS and LR contacts and another with the HIS and SR contacts. These models have the same number of contacts, but the HIS+SR model has a much lower barrier than the HIS+LR model. This is expected from contact order considerations [58]. In addition, too many short loop-length contacts over-stabilize local structure which forms an intermediate. The height of the HIS+LR folding barrier is very similar to that of the FM (Fig. 6c) and we focus on the LR contacts to understand the reasons for the lower folding barrier of HIS.

We select B as the subset of contacts in LR, which have a residue that participates in at least three LR contacts. B has 28 contacts and the folding barrier height of the HIS+B model (Fig. 7c) is almost the same as that of the FM (Fig. 7d). A further analysis of B can be used to identify two clusters of contacts. One of them, M, (10 contacts of residues 5 45 115) gives the myristoyl binding site [43] (Fig. 7a and 7c). The other cluster, A, (13 contacts of residues 20 21 60 61 100 101) is located in the same region as the putative actin binding site [59] (Fig. 7b and 7c). The barrier heights of models HIS+M and HIS+A are much higher than that of HIS. Hybrid models of HIS which include either 10 or 13 extra contacts randomly chosen from the LR set show a much smaller change in barrier height. Thus, it takes the addition of a specific cluster of contacts from the FM to significantly increase the folding barrier height of HIS.

We use the myristoyl binding site to explain the effect of function on the folding of HIS. The binding site is inside the HIS β-barrel. In order to accommodate the myristoyl chain, HIS has evolved a space within its β-barrel that is not present in other β-tretoil proteins (Fig. 8). Upon structural comparison of the HIS to the FM, this space shows up as an absence of contacts between specific residues in the barrel. While folding, HIS has to make fewer contacts and the folding barrier reduces, but the looser packing reduces the structural stability of the fold. This will likely translate into the experimental stability of the protei"n. Thus, the myristoyl binding site demonstrates an entirely different way in which function affects folding.

## Discussion

**The folding-function tradeoff.**

The construction of the FM helps identify structural devThe energy function used iiations in WT proteins which frustrate folding, and which, in turn, are likely to be functional. A WT protein can locally differ from the constructed FM in one of two ways: (a) The WT hThe energy funcThe timescale of protein function puts an upper boundtion used in thas more residues and/or contacts than the FM. An example of this would be a longer loop in the WT which could incorporate an extra secondary structural element like an α-helix or a β-hairpin. This structural element could interact with another protein or another part of the WT in order to function. Extra contacts could also be used to create local order and precisely position residues for binding in the WT. (b) There are locally more residues and/or contacts in the FM. The WT could be smaller to create space for the longer loops of a binding protein or as in HIS the space could be used to bind co-factors. (a) would make the structure of the WT and in turn its folding more complex. As an example, functional loops from distant sites within the protein can together create a binding interface [18]. Such features would increase the complexity of the basic fold as specified in the FM and decrease the folding rate of the WT. On the other hand, WT proteins with (b) would have lower barriers to folding than the FM. In this article, we show examples of (a) in IL-1β and largely (b) in HIS.

The FM shows that function can be built into an “ideal” fold (a well-folding and stable structure) in two ways (Fig. 8): (a) By preserving the folding core and adding new structural elements onto the fold. This is likely to make both structure and folding more complex. (b) By using residues which are part of the folding core to perform function. This is likely to reduce the structural stability of the fold, the barrier to folding and make folding faster. The former is a structural or topological effect and can be classified as topological frustration [31]. The latter is an energetic effect of reducing or destabilizing residues/contacts which were part of the folding core and using them for protein function. This can be classified as energetic frustration [10], [27]–[28]. In a model based only on structure, it is not possible to predict the type of energetic trapping and frustration that arises from non-native interactions that form as the protein folds [10]. The construction of the FM however allows us to predict not only topological frustration but also functional energetic frustration that is present in the folded state of the protein [27]–[28]. In natural proteins, function will likely induce a combination of energetic and topological frustration.

### Residual trapping

Since proteins have to fold on a biologically reasonable timescale but no faster, it is likely that there are residues and contacts in proteins which create minor folding traps but are not functional. Such traps will contribute to the local roughness of the funnel-shaped folding energy landscape [46] but are unlikely to cause large changes in barrier heights or folding routes. We thus do not interpret any change in contact map or sequence length as being functionally relevant unless it causes a change in the folding barrier greater than the roughness of the folding funnel (∼2 k_B_T) [46]. Examples of such residual trapping in the WT proteins studied in this article are given below.

The barrier height of hybrid IL-1β is very similar to that of the FM though the dominant folding route is different (Fig. 4b and 5d). There are contacts specific to IL-1β and the FM which create these differences between the folding routes. But these contacts do not alter the height of the barrier. Since we use that as the measure of folding efficiency, we do not make further predictions about their significance.

Although HIS is shorter than the FM, there are residues in HIS (11 28 29 50 70 99) which do not have corresponding aligned residues in the FM. Of specific interest could be residues 28 and 29 which increase the length of one of the cap loops (seen on the top right of Fig. 6a) and are close to the putative actin binding site [59]. Other than these two residues, the local length increase (accompanied by a length reduction elsewhere) is scattered almost evenly over the HIS cap strands and loops. But Fig. 6c shows that only including all the LR contacts from FM makes the height of the HIS folding barrier almost equal to that of the FM. Thus, we conclude that it is only differences in the contact map and not differences in the backbone between FM and HIS that affect folding.

### Functional regions that can be identified by the FM construction

Structural differences between WT proteins and the FM can occur either because of fewer residues (or contacts) than the FM as in HIS or because of more residues (or contacts) than the FM as in IL-1β. Out of the structural differences found in this article, all except two of those which affect folding are part of known binding regions. Of the two which are not, for the one in HIS, there is some evidence from earlier work that the region identified by the model could be the actin binding site [59].We conclude that, for the β-trefoil fold, structural differences from the FM which affect folding are highly likely to be functional. This method does not need any information about type of function or binding partners to identify functional regions. On the other hand, it does not provide any information about type of function or binding partners either.

This method is unlikely to pick up all functional regions, in particular those where folding and function are both optimal. One can imagine a case where a small surface residue, say an alanine, is enough for packing and folding optimally. Instead a larger residue which has conformational flexibility, say leucine, is present in its place in the protein. Part of the leucine can take the place of the alanine and promote folding while the rest can be co-opted to create a functional region. In this example, the FM will contain only the “alanine” contacts or those relevant to folding and packing. If the leucine does not create any further contacts then it will not be identified by the FM method as being functional. Thus, if there are functional regions in WT proteins where no folding-function tradeoff exists then the FM will not be able to identify them.

The examples of protein function used in this article are those of binding sites, but other types of functional regions are likely to create effects similar to the ones already described here (Fig. 8). As an example, an enzyme active site could create a cavity within a protein in which the ligand binds. This is likely to make folding faster. A nuclear localization sequence on the other hand might be added as an extra secondary structural element and will likely slow folding. The effects of such function on folding need to be quantified by constructing the FMs of appropriate folds such as the enzymatically active TIM barrel fold.

## Conclusions

In this article, we examine the folding-function tradeoff in proteins by constructing a computational model of the “function-less” folding motif (FM) of the β-trefoil fold. The procedure for the construction of the FM is general and can be applied to any fold. We compare the folding of the FM to that of two functional β-trefoil proteins: interleukin-1β (IL-1β) and hisactophilin (HIS). We find that the binding sites in IL-1β decorate the core β-trefoil fold (as seen in the FM), make its structure more complex and slow its folding. In contrast, in HIS, residues which are part of the core β-trefoil fold are reallocated for function. This perturbs packing in the functional regions, reduces the density of contacts and increases the folding rate. Through structural comparison to the FM, we predict that a loop in IL-1β could be of functional significance. We also identify a cluster of residues in HIS that are likely to be part of the actin binding site. Thus, the FM can help identify non-evident functional regions without any input about what that function might be.
